# Level of knowledge and misconceptions about brain concussion in Brazilian adults

**DOI:** 10.1590/0004-282X-ANP-2019-0436

**Published:** 2021-06-23

**Authors:** Amanda Vitória Lacerda De Araújo, Renata Areza-Fegyveres, Carla Cristina Guariglia, Jéssica Natuline Ianof, Regina Maria Baratho, José Luiz Carlos Demario, Rafael Gustavo Sato Watanabe, Renato Anghinah

**Affiliations:** 1 Universidade de São Paulo São Paulo SP Brazil Universidade de São Paulo, Ambulatório de Reabilitação Cognitiva Pós-Trauma Cranioencefálico, São Paulo, SP, Brazil.; 2 Universidade de São Paulo São Paulo SP Brazil Universidade de São Paulo, Centro Dr. Jair Minoto Abe, São Paulo SP, Brazil.

**Keywords:** Brain Concussion, Cerebral Concussion, Health Care, Questionnaires, Concussão Encefálica, Concussão Cerebral, Atenção à Saúde, Questionários

## Abstract

**Background:**

Brain concussion (BC) is seen as a public health priority due to its high incidence and morbidity rate, among thousands of people around the world. There are needs for fast identification, accurate diagnosis and correct management in order to reduce the short and long-term problems relating to BC. Proper knowledge of BC in the population and among clinicians is a critical factor in achieving this.

**Objectives:**

To evaluate the level of self-reported BC knowledge and gaps/misconceptions, and to identify variables correlated with this level.

**Methods:**

A cross-sectional descriptive survey was performed. A Brain Concussion Knowledge Questionnaire (BCKQ) that had been created to capture data was widely distributed. Total scores, domain partial scores and percentages of correct and incorrect answers were calculated to ascertain the level of knowledge relating to BC.

**Results:**

The sample was formed by 1,247 Brazilian adults (age: 41.7±11.8 years). Partial scores of the BCKQ revealed the existence of poor knowledge and misconceptions in all domains of the questionnaire, especially regarding questions about recovery from and management of BC. Moderate correlations between BCKQ scores and professions (p=0.312; P=0.00) or previous brain concussion knowledge (p=0.489; P=0.00) were observed. In a multiple linear regression model, age, profession and sports practice were predictors of BC knowledge.

**Conclusion:**

This first study to analyze the level of BC knowledge in a sample of Brazilian adults suggests that poor knowledge and misconceptions are present. Thus, meaningful and useful information was provided by this study for developing health education programs about BC for the population in order to improve fast diagnosis and correct BC management.

## RESUMO

**Introdução:**

A concussão cerebral (CC) é considerada prioridade em saúde pública devido à sua alta incidência e taxa de morbidade. Há necessidade de identificação rápida, diagnóstico acurado e manejo correto após essas lesões, com objetivo de reduzir problemas de curto e longo prazo. O conhecimento apropriado da população, atletas e de clínicos é primordial para que isto seja alcançado.

**Objetivos:**

Avaliar o nível de conhecimento autorrelatado sobre CC, descrever lacunas/equívocos e identificar variáveis correlacionadas com este nível.

**Métodos:**

Foi realizada uma pesquisa descritiva transversal. O Questionário de Conhecimento sobre Concussão Cerebral (QCCC) criado foi amplamente distribuído. Pontuações totais e parciais em domínios e porcentagens de respostas corretas e incorretas foram calculadas para obter o nível de conhecimento sobre CC.

**Resultados:**

A amostra foi formada por 1.247 brasileiros adultos (41,7±11,8 anos). Pontuações parciais do QCCC revelaram baixo nível de conhecimento, principalmente em questões sobre recuperação e manejo. Observou-se correlações moderadas entre a pontuação no QCCC e a profissão dos participantes (p=0,312, P=0,00) ou conhecimento prévio sobre CC (p=0,489, P=0,00). A regressão linear múltipla mostrou que a idade, profissão e prática de esportes podem ser preditoras do conhecimento sobre CC.

**Conclusão:**

Esse primeiro estudo a analisar o nível de conhecimento sobre CC em uma amostra de adultos brasileiros sugere baixo conhecimento e equívocos sobre esta condição. Assim, informações úteis e significantes foram fornecidas para o desenvolvimento de programas de educação em saúde sobre CC para o público, com objetivo de melhorar a identificação rápida e o manejo correto de concussões.

## INTRODUCTION

Brain concussion (BC) is a type of mild traumatic brain injury (TBI)^1^. It is a complex injury that increasingly is receiving the attention of researchers. BC is seen as a public health priority^2^ due to its high incidence among contact sports players^3^^,^^4^ and the large numbers of cases in the general population^5^^,^^6^, which leads to morbidity in thousands of people worldwide. The United States Centers for Disease Control has estimated that the annual incidence of TBI ranges from 1.6 to 3.8 million cases, including sports-related concussion^7^. In Brazil, Junge et al.^8^ showed that TBI was the second most prevalent injury in football (soccer) during the World Cup in Brazil in 2014. Thus, brain concussions are seen as a public health priority^2^ particularly in countries where contact sports are very common, such as football in Brazil.

A non-penetrating blunt head trauma resulting in functional transient brain disturbance may involve altered mental status, loss of consciousness and post-concussive symptoms, such as headache, dizziness, memory deficits, poor attention or irritability^9^^,^^10^. Therefore, BC is of concern because of the spectrum of potential complications relating to it.

Potential complications involve persistence of post-concussive symptoms for prolonged periods of time (>3 months), and are seen in 10‒15% of concussed individuals^11^^,^^12^^,^^13^. Prolonged post-concussive symptoms are related to impairments of quality of life^14^ and may lead to contact sports players’ retirement^5^^,^^14^. Furthermore, repeated brain concussions have been associated with chronic traumatic encephalopathy in contact sports players^14^. This progressive tauopathy leads to deleterious effects on long-term brain functioning^12^. Moreover, a second impact syndrome leading to catastrophic cerebral edema and death may occur if people suffer a second impact before the first BC has been resolved^15^^,^^16^.

Because of the potential harm caused by BC, and its incidence, there are needs for fast identification, accurate diagnosis and correct management, to reduce the short and long-term problems. Identification of BC depends on symptoms detected through clinical observations and patient self-reporting, or observations by witnesses^5^^,^^10^. Thus, proper knowledge of BC in the population and among sports players and clinicians is a critical factor for improving the identification, reporting and correct management of BC as well as reducing post-concussive sequelae, severe long-term brain function consequences, and death^16^^,^^17^.

Knowledge regarding BC in the general population has been investigated in a range of studies around the world. However, the results have suggested that knowledge or misconceptions about BC is poor among sports players clinicians, and general population^18^^,^^19^^,^^20^. Thus, there is a need for BC health education programs around the world, and efforts to implement such programs are currently underway it^18^^,^^19^^,^^20^. On the other hand, to the best of our knowledge, there are no Brazilian studies on the level of knowledge about BC in Brazil. The lack of evidence about BC knowledge among Brazilians makes it difficult to develop health education programs and, consequently, to prevent BC. Thus, the current study has an important role, consisting of identifying whether there is a lack of knowledge or misconceptions about BC. Furthermore, these data should serve as a basis in the literature for developing future studies that aim to carry out health education programs to alert about the dangers of BC.

Moreover, the present study on BC knowledge levels should highlight problems relating to access to health information and prevention of BC in a population in Brazil, an emerging country. This is particularly important because previous Brazilian studies showed that the estimated incidence of TBI was low^18^, compared with what has been reported in studies conducted in other countries. Moreover, previous Brazilian studies found low rates of brain concussion reported (38‒53%) among TBI cases notified^21^, in comparison with the BC rates of 80‒90% identified^10^^,^^18^ in other studies. Lower incidence and notification rates could be related to poor BC knowledge in the population and, therefore, problems with case identification, thus leading to unreported hospital notifications. Thus, based on previous data^10^^,^^22^, prevention of potentially serious short and long-term consequences relating to BC could be impaired by poor knowledge.

Hence, there is a critical need to develop research evaluating the Brazilian population's BC knowledge in order to identify problems or misconceptions about BC and provide the basis for developing health education programs aimed at prevent the consequences of these shortcomings. The current cross-sectional study was performed (1) to evaluate the level of self-reported BC knowledge in a sample of Brazilian adults; (2) to describe gaps or misconceptions; and (3) to identify variables correlated with BC knowledge levels.

## METHODS

In order to assess the level of BC knowledge in a sample of Brazilian adults, a cross-sectional descriptive survey was conducted. An online questionnaire was distributed between January and August 2018. Ethical approval was obtained from the University of São Paulo Human Research Ethics Committee; all participants provided online informed consent prior to study enrolment; and data were stored and processed anonymously.

### Participants

Participants were selected through an open sampling procedure by means of clusters. Self-administered online surveys were distributed to potential participants randomly. Participants belonging to sports clubs, athletic associations, universities and schools located in São Paulo, Brazil, were recruited. Participants were considered eligible if they were males or females living in Brazil, aged over 18 years and native Portuguese speakers. Invitations to complete the survey were disseminated via e-mail and social media, consisting of an information form that explained the details of the study and invited participation.

### Survey design

Based on a detailed review of the literature, previous surveys3,23 and feedback from two experts in the field of BC (RAF and RA), a pilot version of the structured 31-item Brain Concussion Knowledge Questionnaire (BCKQ) was generated and screened for mistakes. The pilot version was corrected and subsequently applied to a representative sample of Brazilians. Additional adjustments were made to produce the final version of the BCKQ.

Statements for the BCKQ were context-adapted from the previous studies^3^^,^^23^. Additional statements were included with a view to deepening the evaluation of concussion knowledge. The BCKQ was developed on an online platform (Google Forms®).

An online-link survey method was used to improve the applicability of the BCKQ because this allows recruitment of a larger sample and makes it possible for individuals who would be unable to attend a face-to-face meeting, to respond to the survey from their homes^24^. Moreover, instant electronic data archiving makes the data collection more flexible^25^. The BCKQ contained written instructions, an online informed consent form and 31 items designed for self-administration.

The BCKQ was structured into three parts: a demographic section (sex, age, schooling and profession), an experience-related section (sports practice, BC history and previous concussion education) and a brain concussion knowledge section (31 items). These 31 items were divided into four specific domains: identification and causes of BC (six items); functional and neurological consequences of BC (nine items); recovery and management after BC (nine items); and sports-related BC (six items). Each item presents BC facts or misconceptions, in which the participant is able to choose between true or false alternatives. Additionally, the BCKQ has a checklist containing the fourteen most frequent BC signs or symptoms^26^^,^^27^ plus four distractor symptoms. The participants were informed that they should mark all signs/symptoms that they thought were related to BC. No definition of BC was provided for the participants, so that this would not influence the participants’ responses.

All 31 BCKQ items were tabulated to create a total BC knowledge score. Each item of the specific domains was marked as either correct or incorrect. A score of one point was given for the correct answer. Total BC knowledge was calculated by summing the number of correct answers. The total possible BCKQ score is 48 points: this score representing 100% correct answers and, therefore, high or improved knowledge of BC. The total score is formed by the sum of the partial scores from the domains. In the domains of identification and causes of BC and sports-related BC, a score of six points is possible. In the domains of functional and neurological consequences of BC and recovery and management, nine points are possible. The 18-item checklist was scored with one point for each correct sign or symptom (ranging from 0 to 18).

### Statistical analysis

The data were entered into a Microsoft Excel spreadsheet (v. 2010, Microsoft®). Descriptive statistics were calculated to summarize demographics, total BCKQ score, partial scores, percentage of correct and incorrect answers and adherence rate. Normally-distributed data were described as the mean and standard deviation (SD) and non-normal data as the median and percentile. Frequency and percentile were used to describe categorical variables. Our primary outcomes were the total score found through the BCKQ survey, partial scores for each domain and percentage of correct answers. The data were then entered into, and all analyses were performed in, the SPSS software, version 21. Pearson's correlation coefficient (r) was used to examine correlations between the total BCKQ score and age. Correlations between the total BCKQ score and the variables of sex, degree of schooling, type of profession, practicing of sports, previous concussion knowledge and history of concussion were analyzed by means of Spearman's rho (p). All analyses were conducted with the significance level set at p<0.05. Missing data were reported in the Results section and were used to calculate the adherence rate of the BCKQ. A multiple linear regression model was used to determine factors influencing the level of knowledge about BC. The independent variables included in the regression model were age, sex, degree of schooling, type of profession and practicing of sports and its frequency.

## RESULTS

The BCKQ survey was completed by 1247 Brazilian adults (mean age 41.7±11.8 years), living in São Paulo. The demographics of the participants are listed in Table 1.

**Table 1:** Demographic characteristics of the study sample (n=1,247).

Demographic characteristics		Frequency (%)
Sex (n=1,247)		
	Female	1048 (84)
	Male	199 (16)
Age in years (n=1,247)		
	<20	9 (0.7)
	20–29	165 (13.2)
	30–39	435 (34.9)
	40–49	313 (25)
	50–59	210 (16.8)
	60–69	103 (8.3)
	>70	12 (1)
Education level (n=1,247)		
	Elementary school	6 (4.8)
	High school graduate	121 (9.8)
	Bachelor's degree or higher	1120 (89.8)
Profession		
	Fields of healthcare or sports	284 (23)
	Other areas	963 (77)
Type of sports (n=1,214)		
	Contact sports	61 (5)
	Non-contact sports	704 (58)
	None	449 (37)
Level of sports practice (n=1,239)		
	Professional	21 (1.8)
	Semi-professional	49 (4)
	Recreational	703 (56.7)
	None	466 (37.6)
Sports frequency		
	None	449 (36)
	Once every 15 days	78 (6.3)
	Once or twice a week	288 (23.1)
	Three or four times a week	313 (25.1)
	Five or six times a week	69 (5.5)
	Daily	50 (4)

Table 2 summarizes the data on previous BC knowledge and history of BC. A total of 56% of the participants (n=700) reported having previous knowledge about BC. However, a high number of people reported the alternative “I do not know or I'm not sure what brain concussion is” (44%; n=547). These data highlight the absence of knowledge about BC in a high percentage of the sample and should be considered concerning. Participants who checked the option “I do not know or I'm not sure what brain concussion is” did not proceed to the next section of the questionnaire. Thus, the BCKQ total and partial scores are presented based on a sample of 700 participants.

**Table 2:** Information on previous knowledge of concussion or history of concussion (n=1,247).

Questions and alternatives		Frequency (%)
Have you heard of concussion? (n=1,247)		
	Yes	701 (56)
	No	546 (44)
What is your source of knowledge about concussion? (n=1,014)		
	Healthcare professional	268 (38)
	Television, newspaper or magazine	272 (39)
	Internet	189 (27)
	Coach or team coach	25 (4)
	Congress, conference or scientific meeting	51 (7)
	Others	209 (30)
Have you ever had concussion or do you live with someone who has had concussion? (n=1,365*)		
	I have never had concussion	635 (51)
	Yes, I have already had concussion	62 (5)
	Yes, I have a family member or friend who had concussion	126 (10)
	I do not know or am not sure what concussion is	542 (44)
	Not available	7 (1)

* Question with multiple possible answers.

**Figure 1:**
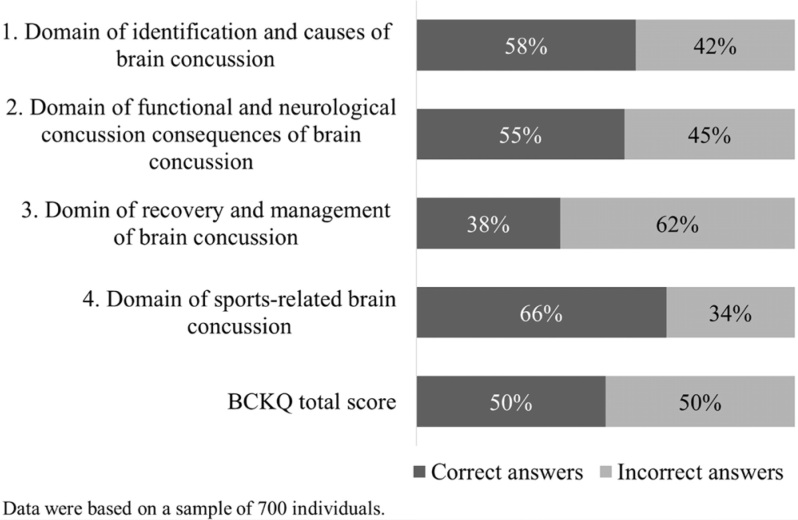
Percentages of correct and incorrect answers in each specific domain of the Brain Concussion Knowledge Questionnaire, among the sample of Brazilian adults (n=700).

Knowledge gaps and misconceptions were identified through the BCKQ total score. The mean total BCKQ score was 24±10.3 points, corresponding to a correct-answer rate of 50%. Thus, poor BC knowledge was observed. Based on the partial scores for each domain, specific larger gaps or misconceptions could be seen in the domain of recovery and management after BC, with the lowest mean score (3.5±1.8 points) and lowest rate of correct answers (38%) (Figure 1). However, all the domains had low scores. The mean score in the domains of identification and causes of BC (3.5±1.5 points) and functional and neurological consequences of BC (5±2 points) represented correct-answer rates of 58 and 55%, respectively. The domain of sports-related BC had a mean score of 4±1.4 points and a correct-answer rate of 66%.

**Figure 2:**
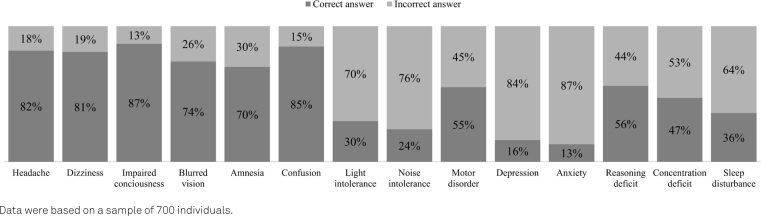
Percentages of signs and symptoms most commonly correlated with brain concussion, according to the responses among the sample of Brazilians adults, in the Brain Concussion Knowledge Questionnaire (n=700).

In the question about the main signs or symptoms of BC, the participants’ mean score for the 18-item checklist was 7±3.3 points, corresponding to a correct-answer rate of 38%. Figure 2 shows the percentage of correct and incorrect answers. Anxiety (13%), depression (15%), noise intolerance (21%), light intolerance (26%) and sleep disturbance (34%) had the lowest rates of correct answers (Figure 2).

Table 3 show detailed information about the percentages of the answers to each BCKQ statement. Alarming gaps and misconceptions were observed. There was a high rate of incorrect answers (87%) to the statement “the only way to be sure that a person has suffered brain damage from a concussion is by cranial tomography or magnetic resonance imaging of the skull”. Sixty-five percent believed that “emotional problems after brain concussion are usually not related to brain damage”. Similarly, 60% chose an incorrect answer when asked if “a brain concussion can make a person feel depressed, hopeless and sad”. High rates of incorrect answers were also observed regarding the statements “it is easy to tell if a person has had brain damage from a concussion because of the way a person behaves” (56%) and “most people who suffer a brain concussion do not realize the effect of the injury on their behavior and reasoning” (55%). Also, most of the participants (69%) did not know that “a person may die if a second brain concussion occurs before recovery from an earlier concussion”. Impairment of the capacity to learn new things was also a knowledge gap for 358 of the participants (52%).

**Table 3:** Knowledge gaps identified for each statement in specific domains of the Brain Concussion Knowledge Questionnaire, according to the answers of the Brazilian sample (n=700).

BCKQ (n=700) Statements (correct answer)		Frequency (%)		
		True	False	NA
Domain of identification and causes of brain concussion				
	1. Brain concussion can cause brain damage even if the person has not fainted (T)	580 (82)	120 (18)	0
	2. A person needs to have passed out to be diagnosed with brain concussion (F)	154 (23)	544 (77)	2
	3. The only way to tell whether someone has suffered brain damage from concussion is by mean of cranial tomography or magnetic resonance imaging (F)	608 (87)*	91 (13)	1
	4. Brain concussion can only occur if there is a direct hit to the head (F)	233 (34)	465 (66)	2
	5. An impact on the body that causes rapid acceleration and deceleration of the head can cause brain damage even if there is no direct blow to the head (T)	528 (75)	172 (25)	0
	6. It is easy to tell whether a person has brain damage from concussion from the way the person looks or behaves (F)	391 (56)*	309 (44)	0
Domain of functional and neurological consequences of brain concussion				
	7. Brain concussion is harmless and never results in long-term problems or brain damage (F)	196 (28)	504 (72)	0
	8. A little brain damage does not matter, as people only use a small part of their brains anyway (F)	92 (13)	607 (87)	1
	9. Most people who suffer brain concussion do not realize the effect of concussion on their behavior and reasoning (T)	310 (45)	388 (55)*	2
	10. A person may die if a second brain concussion occurs before recovery from a previous one (T)	223 (41)	473 (69)*	4
	11. Brain concussion can make a person feel depressed, hopeless and sad (T)	278 (40)	422 (60)*	0
	12. Emotional problems after brain concussion are usually not related to brain damage (F)	453 (65)*	245 (35)	2
	13. A person who has suffered brain concussion may have trouble recalling facts before the concussion (T)	547 (78)	151 (22)	2
	14. Multiple concussions over the course of life can give rise to brain problems (T)	518 (74)	180 (26)	2
	15. A person who has suffered brain concussion may have trouble learning new things (T)	330 (48)	368 (52)*	2
Domain of recovery and management of brain concussion				
	16. Sometimes a second blow to the head can help a person remember things that were forgotten (F)	273 (40)	425 (60)	2
	17. Once a recovering person feels normal, the recovery process is complete (F)	197 (29)	501 (71)	2
	18. Complete recovery from brain concussion is not possible, no matter how badly the person wants to recover (F)	362 (52)*	337 (48)	1
	19. How quickly a person recovers from brain concussion depends mainly on how hard they work on recovery (F)	374 (54)*	324 (46)	2
	20. It is recommended to rest and remain inactive during recovery from brain concussion (F)	521 (76)*	178 (24)	1
	21. After 10 days the symptoms of brain concussion are usually completely gone (T)	65 (8)	634 (91)*	1
	22. A person who has not recovered from brain concussion is less able to withstand a second blow to the head (T)	297 (42)	402 (58)*	1
	23. The most appropriate way to evaluate the progress of recovery after brain concussion is by asking the person who was concussed how they feel (T)	101 (14)	596 (86)*	3
	24. Most people wake up quickly and undamaged after being knocked unconscious by a blow to the head (T)	165 (24)	531 (76)*	4
Domain of sports-related brain concussion				
	25. In sports, brain concussion rarely happens (F)	70 (10)	628 (90)	2
	26. Athletes who have had one brain concussion are more susceptible to having another (T)	178 (26)	517 (74)*	5
	27. Athletes who practice contact sports are at greater risk of brain concussion (T)	560 (80)	137 (20)	3
	28. A soccer player who has suffered brain concussion may continue to play as long as they feels good (F)	385 (55)*	313 (45)	2
	29. A soccer player who has suffered brain concussion during a workout can return to the next ball practice as long as they feel good (F)	401 (57)*	295 (43)	4
	30. A soccer player who felt dizzy after suffering brain concussion and returned to dizziness when training should continue training until the dizziness improves (F)	173 (25)	523 (75)	4

BCKQ: Brain Concussion Knowledge Questionnaire; F: false; T: true.

Fifty-two percent of the participants (n=362) believed that “recovery from a brain concussion does not occur”. Most of the participants (91%) also did not know that the symptoms of a brain concussion pass after 10 days. Equally, 74% of the sample (n=517) presented incorrect answers when asked if “sports players who had a brain concussion would be more likely to have another”. Fifty-five percent (n=385) thought that a sports player who had suffered a BC could continue playing if he or she felt good. Likewise, 57% of the sample believed that a “soccer player who had suffered a brain concussion could return to the next ball practice if he or she felt well”.

The BCKQ adherence rate remained high for most questions, with a rate of missing answers<1%. Moderate positive correlations between BCKQ total scores and professions linked to the fields of healthcare or sports were observed (p=0.312; P=0.00). Similarly, the BCKQ total score also showed a moderate positive correlation with previous BC knowledge (p=0.489; P=0.00). Only poor correlations were found in relation to sex (p=0.096; P=0.00), age (r=0.2; p=0.00) and degree of schooling (p=0.106; P=0.00). No correlations were observed for the other variables analyzed.

Multiple linear regression was performed on the data relating to 700 participants (Table 4). This showed that age was significantly related to the level of BC knowledge. Thus, for every one-year increase in age, the participants tended to have a BCKQ score that was 0.15 points lower. Type of profession, which was categorized as healthcare-related, sports-related or unrelated to either of the preceding categories, was also a predictor. Thus, participants in healthcare-related professions tended to have higher BCKQ scores. Participants in sports-related professions had BCKQ scores that were 7.03 points lower. This was an alarming result because sports professionals are close to individuals who are more likely to suffer a BC. Furthermore, participants who practiced sports had BCKQ scores that were 1.82 points lower than those of participants who did not. Curiously, regression analysis showed that participants who practiced sports daily had BCKQ scores that were 4.01 points lower (Table 4). Since the individuals who played sports daily in our sample were athletes, this is a worrying result. No interaction occurred between the other variables included in the model.

**Table 4:** Multiple linear regression model to predict variables relating to brain concussion knowledge (n=700).

Variables		Estimate	SE	p-value
Age (years)		-0.15	0.03	<0.001*
Sex (female)		1.20	0.93	0.195
Profession				
	Healthcare (reference category)	1.00	-	-
	Sports-related	-7.03	2.37	0.003*
	Not related to healthcare or sports	-7.32	0.84	<0.001*
Degree of schooling				
	Elementary school (reference category)	1.00	-	-
	High school graduate	6.19	6.95	0.373
	Bachelor's degree or higher	6.59	6.85	0.336
	Sport practice (yes)	1.82	0.78	0.021*
Sports frequency				
	None (reference category)	1.00	-	-
	Once every 15 days	-1.58	1.55	0.309
	Once or twice a week	-1.84	0.97	0.058
	Three or four times a week	-1.70	0.98	0.084
	Five or six times a week	-1.00	1.68	0.551
	Daily	-4.01	1.92	0.037*

SE: standard error;

* p<0.05.

## DISCUSSION

To the best of our knowledge, this is the first study to examine the level of BC knowledge among Brazilian adults. The results from this cross-sectional descriptive survey provide evidence of poor BC knowledge and a moderate rate of misconceptions in a sample of Brazilians with a high level of schooling. Our findings showed that a high proportion of the sample did not know what BC is (44%). These data highlight the absence of knowledge about BC in a high percentage of the sample and should be considered concerning.

Moreover, there was poor knowledge in all BCKQ domains, i.e., the participants had impaired knowledge of how to identify a BC and what causes it, the functional and neurological consequences of BC, the recovery and correct management after BC and the recovery after sports-related BC. These findings are supported by the fact that many of the participants stated that they did not know what a BC is. On the other hand, a proportion of the participants stated that they had previous BC knowledge from healthcare professionals, the internet or television, newspapers or magazines. Thus, our findings show that when the participants had previous BC information, the transfer of knowledge may have been impaired, thus resulting in gaps or misconceptions. Furthermore, an additional concern was noted through multiple linear regression, considering that playing sports daily and having sports-related professions were predictive variables for lower scores in the BCKQ.

### Brain concussion knowledge

The BC knowledge scores varied depending on the BCKQ domain. For instance, there was a higher number of incorrect answers in relation to statements about recovery and management after BC. This finding is worrying because a lack of adequate management and recovery may expose people to more serious consequences, such as post-concussion syndrome9,10, chronic traumatic encephalopathy15 or second impact syndrome16. To prevent the range of potential consequences of a BC, the Brazilian population needs to become familiar with the various aspects of BC management and recovery. Previous studies also found that there was limited knowledge regarding the recovery and management of people with BC^3^^,^^28^.

Similarly, a high number of incorrect answers were observed regarding common signs or symptoms of BC. In our study, people had higher knowledge of post-concussive symptoms such as confusion, headache, impaired consciousness, dizziness, blurred vision and amnesia. However, there were very low numbers of correct answers in relation to behavioral symptoms, light or noise intolerance and sleep disturbance. Previous studies also showed limited knowledge regarding post-concussive symptoms in the general population^29^^,^^30^. Light or noise intolerance was also poorly identified in a study by Knollman-Potter et al.^31^. Data from Waltzman et al.^32^ showed that a small portion of their participants correctly chose sleep disturbance as a common post-concussive symptom. In agreement with our findings, studies have demonstrated that people tend to be familiar with certain BC symptoms, such as headache, dizziness and impaired consciousness^33^^,^^34^. Behavioral symptoms are less correlated to a BC by the general population^31^.

Overall, previous studies identified poor levels of BC knowledge in various aspects such as post-concussive symptoms, identification, recovery and management after the BC^3^^,^^17^^,^^19^^,^^20^^,^^34^^,^^35^. For example, McKinlay et al.^21^ reported that there was significant uncertainty in the general population about what a BC is and how it should be managed. Other studies also reported these findings^29^^,^^32^. Our results were generally consistent with these previous studies.

Interestingly, the first international study^36^ about BC knowledge showed that a significant proportion of their sample believed that a second blow to the head could help memory recover, which also was shown in our study. A recent study showed that the proportion of people who considered this statement correct was only 10%^3^. Our results in this regard are only concordant with those of a study conducted in 1988. Thus, there is concern about this information, and it highlights the significant breadth of misconceptions found in our study.

Other specific findings from previous studies are concordant with our results. One study^3^ showed that a high number of participants rejected the statement regarding the increased likelihood of a second blow to the head in sports. The percentage of incorrect answers to this statement was also high in our study. Similarly, these authors^3^ indicated that a substantial number of participants considered that the most appropriate way to evaluate the recovery would be to ask individuals who had become concussed how they felt. The same was observed in our sample. In comparing our findings with those of the study by Weber and Edwards^3^, on which our questionnaire was based, the level of BC knowledge in our sample was significantly lower. Thus, our data support the data in the current literature and show that Brazilian adults with high schooling levels had poor knowledge about BC. These findings highlight the urgency of health education regarding BC in Brazil. Studies have indicated that implementation of health education programs for the general population have led to gains in knowledge regarding the symptoms of BC, identification of this condition and correct recovery from it^18^^,^^37^^,^^38^.

Improvements of knowledge about BC in the general population may increase the frequency of symptom reporting and demands for treatment^39^, as well as helping to prevent a range of complications and sequelae^18^ relating to BC. In the light of the results from these previous studies, an increasing number of health education programs aimed at various sectors of the population have emerged in other countries^18^^,^^37^^,^^38^^,^^39^. Unfortunately, this scenario has not been observed in Brazil.

Hence, the current study highlights the need for an approach to health education for the general population regarding prevention of BC, which would reduce the potentially serious short and long-term consequences of this injury. The results highlight evidence of poor knowledge and misconceptions about BC, which may be related to impaired prevention plus a high rate of unreported BC at healthcare services in Brazil^10^^,^^18^. Furthermore, our results serve as a basis for developing future studies that aim to develop health education programs for the general population or to investigate specific aspects of the poor knowledge and misconceptions found in this study.

Regarding health education programs aimed at the general population, previous studies have shown that as more information about the etiology, management and sequelae of BC has emerged, awareness about the importance of safe concussion management has improved^17^^,^^18^^,^^31^^,^^37^^,^^38^^,^^39^. This has been demonstrated through improvements in some concussion-related knowledge over time38. Thus, understanding the knowledge gaps and misconceptions contributes to formulating strategies that are needed to specifically address these problems^29^.

Education programs should ideally promote active engagement of patients and improve the ability to apply previous healthcare knowledge in situations of everyday life^31^. The approaches used need to include multiple parts of society, such as the general community, parents, caregivers, athletes and healthcare professionals^39^. Furthermore, dissemination of information should include government resources, campaigns and structured programs within public healthcare services^31^. To educate the general population about BC, the educational methods and materials used should be focused on websites, flyers or lectures^17^^,^^31^^,^^39^. However, before BC education programs are established, studies should be conducted to ascertain the most effective means of disseminating information to the general population.

### Study limitations and future research

There are several limitations to this study. First, despite the authors’ attempt to obtain a diversified sample of Brazilian adults, the final sample was composed of a higher number of females, people with high schooling levels and non-athletes. This sample composition may limit the ability to generalize the results from our study. Second, use of an online survey to reach a wider range of participants may introduce the inability to verify whether participants answered the questions without help from other sources of knowledge.

For future research, the aim should be to have a probabilistic sample that is also homogeneous, i.e., with a balance of factors such as sex, age, profession, socioeconomic status, education level and exercise practice level. Moreover, studies using interviews, comprehensive methodologies, qualitative research or open-ended questions may help to understand patterns of responses, difficulties in interpretation or misconceptions of understanding of the survey. Larger studies aiming to ascertain the variables that influence knowledge of concussion should be conducted. In addition, future studies investigating the effectiveness of using health education programs about BC are strongly recommended.

In conclusion, in this first study to examine the level of BC knowledge among Brazilian adults, the findings showed that BC knowledge was poor. This mirrors previous studies. Our study provided very useful information about BC knowledge among Brazilian adults and demonstrated the need for health education programs about BC. Health education programs can improve the knowledge of the population and should help to increase rapid identification and correct management of BC. Larger-scale research is needed to investigate the BC knowledge among groups of Brazilians such as healthcare and sports professionals. Variables that influence BC knowledge should be investigated in larger samples in order to understand what the main focus of health educational programs should be.
